# Construction of a genetic AND gate under a new standard for assembly of genetic parts

**DOI:** 10.1186/1471-2164-11-S4-S16

**Published:** 2010-12-02

**Authors:** Shotaro Ayukawa, Akio Kobayashi, Yusaku Nakashima, Hidemasa Takagi, Shogo Hamada, Masahiko Uchiyama, Katsuyuki Yugi, Satoshi Murata, Yasubumi Sakakibara, Masami Hagiya, Masayuki Yamamura, Daisuke Kiga

**Affiliations:** 1 Department of Computational Intelligence and Systems Science, Interdisciplinary Graduate School of Science and Engineering, Tokyo Institute of Technology, 4259 Nagatsuta-cho, Midori-ku, Yokohama, Kanagawa 226-8503, Japan; 2 Department of Biosciences and Informatics, Keio University, 3-14-1 Hiyoshi, Kohoku-ku, Yokohama, Kanagawa 223-8522, Japan; 3 Department of Bioengineering and Robotics, Tohoku University, 6-6-01 Aoba-yama, Sendai 980-8579, Japan; 4 Department of Computer Science, Graduate School of Information Science and Technology, University of Tokyo, 7-3-1 Hongo, Bunkyo-ku, Tokyo 113-8656, Japan; 5 PRESTO, JST, 4-1-8 Honcho, Kawaguchi-shi, Saitama 332-0012, Japan; 6 Present address: Department of Biophysics and Biochemistry, Graduate School of Science, University of Tokyo, 7-3-1 Hongo, Bunkyo-ku, Tokyo 113-0033, Japan

## Abstract

**Background:**

Appropriate regulation of respective gene expressions is a bottleneck for the realization of artificial biological systems inside living cells. The modification of several promoter sequences is required to achieve appropriate regulation of the systems. However, a time-consuming process is required for the insertion of an operator, a binding site of a protein for gene expression, to the gene regulatory region of a plasmid. Thus, a standardized method for integrating operator sequences to the regulatory region of a plasmid is required.

**Results:**

We developed a standardized method for integrating operator sequences to the regulatory region of a plasmid and constructed a synthetic promoter that functions as a genetic AND gate. By standardizing the regulatory region of a plasmid and the operator parts, we established a platform for modular assembly of the operator parts. Moreover, by assembling two different operator parts on the regulatory region, we constructed a regulatory device with an AND gate function.

**Conclusions:**

We implemented a new standard to assemble operator parts for construction of functional genetic logic gates. The logic gates at the molecular scale have important implications for reprogramming cellular behavior.

## Background

The development of synthetic biology promotes the construction of artificial genetic systems with desired functions inside living cells [1,2]. The bottleneck in the realization of a functioning system is the appropriate regulation of gene expression [3]. Thus far, many studies in synthetic biology have seen difficulties in regulating all genes in the system to work collaboratively. In order to construct an efficient biosynthetic pathway of a precursor anti-malaria drug, the regulation of several gene expressions inside *Saccharomyces cerevisiae* had to be modified in addition to the introduction of exogenous genes [4]. This and other studies using similar modifications indicate that well-designed gene regulation is important to constructing a well-functioning genetic system [5,6].

The engineering of promoters is an effective method for the fine regulation of gene expression for realization of the desired function. Most promoters contain a -35/-10 sequence where an RNA polymerase binds. Also, some promoters contain regulatory protein-binding sites called operators. By changing the strength of the -35/-10 sequence and the operator sequence arrangement, the promoter for the appropriate regulation of gene expression can be constructed.

Regulation assemblies are composed of many types of DNA units. Some of the DNA units are small pieces of DNA containing a protein-binding site. Assembling these DNA units allows for preparation of a synthetic promoter library from which genetic logic gates have been isolated [7]. Another type of DNA unit is a large fragment of DNA containing a protein coding sequence. By assembling several DNA units containing promoter sequences and DNA units containing coding sequences of regulatory proteins, a genetic toggle switch and a repressilator were constructed inside *Escherichia coli*[8,9].

Rational design is one approach to establishing a regulation assembly of DNA units. For example, a synthetic promoter integrating input of arabinose and Isopropyl β-D-1-thiogalactopyranoside (IPTG) was designed by allocation of operators for two different regulatory proteins, AraC and LacI [10]. With a different design, Anderson *et al.* constructed an AND gate based on an interaction between nonsense suppressor tRNA and mRNA with an internal stop codon [11]. For construction of each variation of these assemblies, variation-specific DNA construction was required.

BioBricks were developed as standard DNA parts for construction of designed biological systems [12-16]. For construction of genetic circuits, BioBricks include several classes of genetic parts: ribosome-binding site, protein-coding sequence, protein-binding site, and -35/-10 sequence. Furthermore, the BioBricks parts can be combined laterally because of the standardization of parts, which are standardized by introduction of two sets of restriction sites. The BioBricks standard allows repeating attachments of new parts to the foreside and backside of the already connected parts. This standardization, however, does not allow insertion of parts between already connected parts even though such an insertion is important for modification of a prototype genetic assembly.

In this study, by modifying BioBricks standardization, we developed a new standardization that allows for insertion of parts into an established assembly of other parts and constructed a new genetic multi-input regulation assembly. In addition to the sets of restriction sites in BioBricks, we used another set of restriction sites for the new standardized parts assembly. The new standardization allows for insertion of parts between two pre-connected parts. In fact, by introducing operator parts into the regulatory region of a plasmid that already had allocated another species of an operator part, we constructed a new promoter integrating the inputs of two different inducers into a transcription output. The new assembly of operator parts can also be called a genetic AND gate.

## Results

### Design of a genetic AND gate

**Figure 1:**
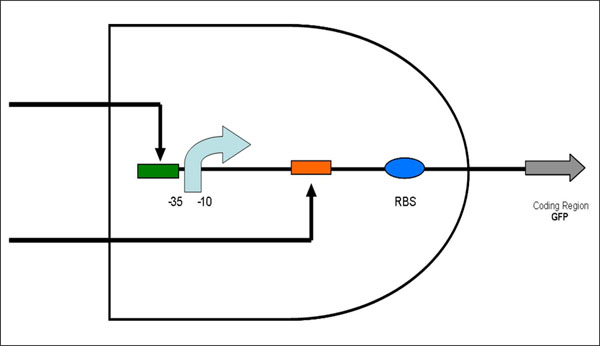
**Scheme of a genetic AND gate regulated by transcription factors.** Assembly of two different operator parts forms a device that functions as a genetic AND gate. This AND gate integrates inputs of two different inducers into a transcription output. The pale blue arrow represents a -35/-10 sequence. The green rectangle represents an operator part inserted into the upstream of the -35/-10 sequence. The orange rectangle represents an operator part inserted into the downstream of the -35/-10 sequence. The black arrows pointing the operators represent the environmental inputs. The blue ellipse represents a ribosome-binding site. The gray arrow represents a coding region of a protein whose transcription is turned on as an output.

An AND gate integrates two inputs into an output. The genetic AND gate we designed works based on the general regulation mechanism of transcription in bacteria. The output is the transcription of a gene which is dually regulated by the inputs of two small molecules. Each of the small molecules, called inducers, controls the binding of the corresponding regulatory protein to the specific binding site on DNA. We call a DNA containing a protein-binding site an operator part. By inserting two different operator parts into the regulatory region of a gene on a plasmid, we designed a regulatory device that functions as an AND gate (Figure 1). In the genetic AND gate, only when both inducers are added as inputs, transcription of the gene is turned on as an output.

### Construction of a standardized platform of the regulatory region

**Figure 2:**
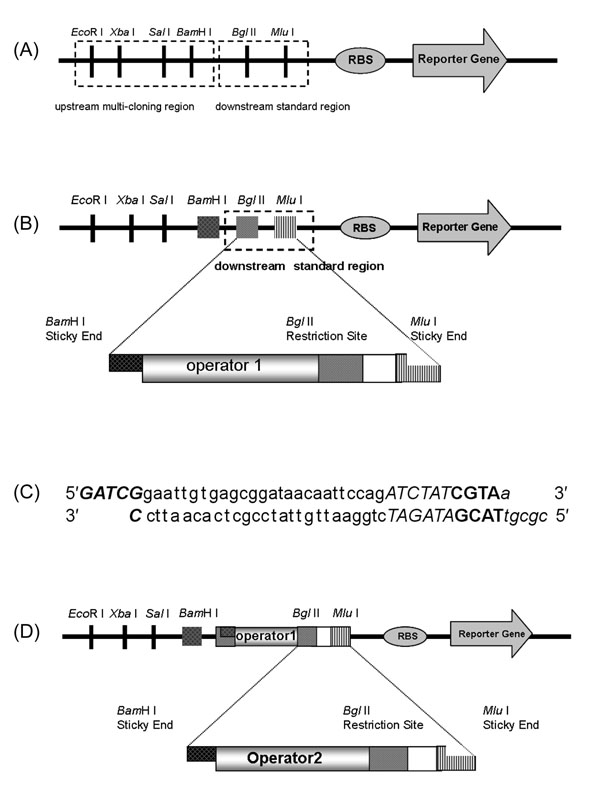
**Standardization of the regulatory region and operator parts.** (A) The regulatory region of a plasmid was divided into the upstream multi-cloning region and the downstream standard region for standardization of the assembly. The upstream multi-cloning region contains the *Eco*R I, *Xba* I, *Sal* I and *Bam*H I restriction sites. The downstream standard region contains the *Bgl* II and *Mlu* I restriction sites. The operator part with a -35/-10 sequence is inserted into the upstream multi-cloning region, while that without a -35/-10 sequence is inserted into the downstream standard region. The sets of restriction sites allow insertion of the operator parts between the pre-connected parts. (B) The operator sequence is flanked by the sticky ends of *Bam*H I and *Mlu* I in the operator part for the downstream standard region. The operator parts are inserted to the reporter plasmid digested with *Bgl* II and *Mlu* I. The *Bam*H I sticky end of the operator part comes together with the *Bgl* II sticky ends of the plasmid. (C) The LacI operator part was designed as a downstream part. The *Bam*H I sticky end (***Bold & Italic***) is added to the left side of the LacI operator sequence (small letters). The *Bgl* II restriction site (*Italic*), spacer (**Bold**), and *Mlu* I sticky end (*small letters & Italic*) are added on the right side of the LacI operator. (D) Although the original *Bgl* II site on the reporter plasmids disappears by the insertion of the operator part, the *Bgl* II site contained on the inserted part works as a new *Bgl* II site of the plasmid. This recovery of *Bgl* II site allows repeating insertions of the next operator parts.

For the systematic assembly of operator parts, the regulatory region of a reporter *gfp* gene on pSB-GFP was standardized by dividing the region into two sub-regions: the upstream multi-cloning region and the downstream standard region (Figure 2A). The upstream multi-cloning region requires an RNA polymerase-binding part called the -35/-10 sequence. Some RNA polymerase-binding parts also accommodate an operator part. The downstream standard region was designed to accommodate multiple operator parts through the following procedures of repeating insertions of the parts into the region upstream of the ribosome-binding site. Corresponding to the *Bgl* II and *Mlu* I restriction sites of the downstream region, the operator part has a *Bam*H I sticky end, a *Bgl* II restriction site, and a *Mlu* I sticky end (Figures 2B and 2C). Because the sticky ends of *Bgl* II and *Bam*H I are compatible, the operator part can be ligated between the *Bgl* II and *Mlu* I sites on the plasmid. Even though the first insertion abolishes the original *Bgl* II restriction site on the plasmid, the operator part has an additional *Bgl* II site that functions as the new restriction site of the downstream standard region (Figure 2D). Thus, the downstream standard region can accommodate multiple operator parts. The assembling of operator parts with different binding sequences in the upstream and/or downstream regions of a plasmid generates a genetic device that integrates input of different inducers into a transcription output.

### Construction of a gate regulated by Lacl repressors

**Figure 3:**
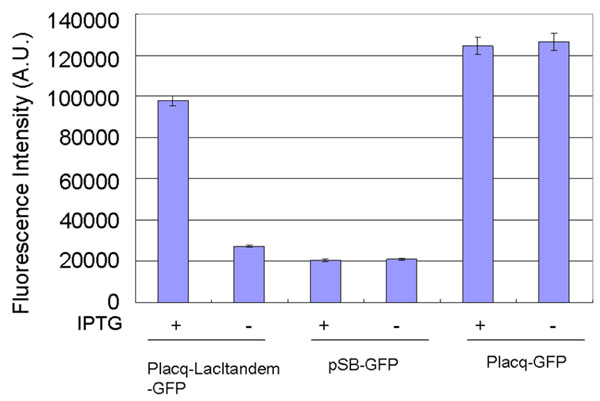
**Reporter assays of Placq-Lacltandem-GFP.** The results show the changes in the fluorescence intensities of the cultures with pSB-GFP, Placq-GFP, or Placq-Lacltandem-GFP dependent on the input of 1 mM IPTG. Cells were cultured for 8 hours after the induction. In the presence of IPTG, the culture with Placq-Lacltandem-GFP showed 3.5-fold higher fluorescence intensity than that in the absence of IPTG. The culture with pSB-GFP, which does not express GFP, was used as a negative control. The culture with Placq-GFP, which constitutively expresses GFP, was used as a positive control. The assays were performed in triplicate. Error bars indicate the standard deviation.

By inserting two operator parts containing a Lacl-binding sequence, a device that generates GFP expression dependent on the input of IPTG input was constructed. First, as a plasmid expressing GFP constitutively, we constructed Placq-GFP by inserting a constitutive promoter part, Placq, into the upstream multi-cloning region of pSB-GFP, a plasmid containing a promoterless *gfp* gene. Based on the standardization method described above, we synthesized a Lacl operator part and inserted it into the downstream standard region of Placq-GFP to construct the plasmid named Placq-LacI-GFP. Since the natural lac promoter contains two lac operator sites on the downstream region, we inserted an additional LacI operator part into the downstream standard region. The standardization allowed additional insertion of the operator part with the same procedure as the first insertion. We named the constructed plasmid Placq-Lacltandem-GFP. Introducing this plasmid into a LacI-expressing *E.coli* strain DH5αLacI, we measured the gene regulation dependent on the LacI operators with the reporter assay using a fluorometer (Figure 3). In the presence of IPTG, the culture with Placq-Lacltandem-GFP showed 3.5-fold higher fluorescence intensity than that in the absence of IPTG. In the absence of IPTG, the culture with Placq-Lacltandem-GFP showed the background-fluorescence intensity generated by promoterless pSB-GFP.

### Construction of a gate regulated by a LuxR activator

By inserting an operator part containing a LuxR-binding site, we constructed another plasmid named PLuxR-GFP, which generates GFP output dependent on the input of N(β-ketocaproyl)-DL-homoserine lactone (AHL). The LuxR operator part is composed of a LuxR expression cassette, a LuxR operator sequence and a weak -35/ -10 sequence called luxpR, which exist in the natural lux operon [17]. In the presence of AHL, a LuxR protein binds to the LuxR operator and activates the transcription.

**Figure 4:**
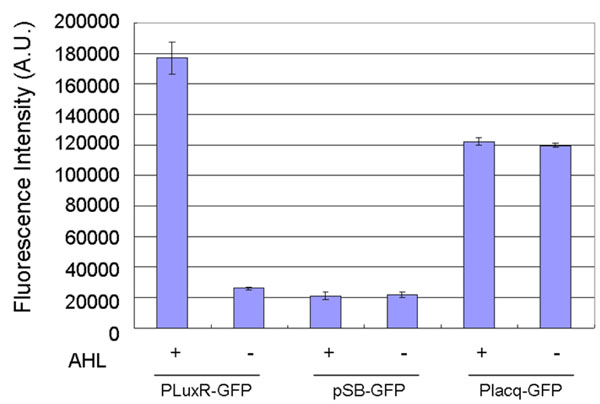
**Reporter assays of PLuxR-GFP.** The results show the changes in the fluorescence intensities of the cultures with pSB-GFP, Placq-GFP, or PLuxR-GFP dependent on the input of 4.5 nM AHL. Cells were cultured for 8 hours after the induction. In the presence of AHL, the culture with PLuxR-GFP showed 6.8-fold higher fluorescence intensity than that in the absence of AHL. The culture with pSB-GFP, which does not express GFP, was used as a negative control. The culture with Placq-GFP, which constitutively expresses GFP, was used as a positive control. The assays were performed in triplicate. Error bars indicate the standard deviation.

Introducing the plasmid PLuxR-GFP into DH5αLacI, we measured the gene regulation dependent on the LuxR operator with the reporter assay using a fluorometer (Figure 4). In the presence of AHL, the culture with PLuxR-GFP showed 6.8-fold higher fluorescence intensity than that in the absence of AHL. In the absence of AHL, the culture with PLuxR-GFP showed the background-fluorescence intensity generated by promoterless pSB-GFP.

### Construction of a genetic AND gate by assembly of operator parts

**Figure 5:**
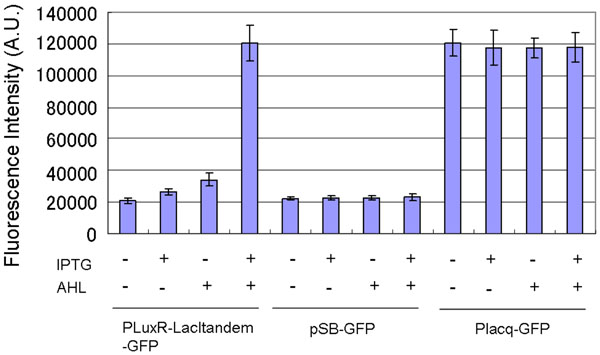
**Reporter assays of PLux-Lacltandem GFP.** The results show the changes in the fluorescence intensities of the cultures with pSB-GFP, Placq-GFP, or PLuxR-LacItandem-GFP dependent on the input of 1 mM IPTG and 4.5 nM AHL. Cells were cultured for 8 hours after the induction. In the culture with PLuxR-LacItandem-GFP, the GFP expression was turned on in the presence of both 1 mM IPTG and 4.5 nM AHL. When neither of the inducers was added, GFP expression was turned off. In the presence of the both inducers, culture with PLuxR-Lacltandem-GFP showed 5.7-fold higher fluorescence intensity than that in the absence of the both inducers. The culture with pSB-GFP, which does not express GFP, was used as a negative control. The culture with Placq-GFP, which constitutively expresses GFP, was used as a positive control. The assays were performed in triplicate. Error bars indicate the standard deviation.

We then constructed a genetic AND gate by assembling two Lacl-operator parts and a LuxR-operator part on one regulatory region as designed. The two LacI-operator parts were inserted sequentially into the downstream-standard region of PLuxR-GFP plasmid with the same procedure as that for construction of Placq-Lacltandem-GFP. The constructed plasmid was named PLuxR-LacItandem-GFP. Introducing this plasmid into DH5αLacI, we measured the regulation of GFP expression dependent on the combination of the LacI operators and the LuxR operator. In the absence of the both inducers, the culture with PLuxR-LacItandem-GFP showed the background- fluorescence intensity generated by promoterless pSB-GFP (Figure 5). The presence of either IPTG or AHL alone had little effect on increasing the fluorescence intensity. In the presence of both inducers, the culture showed 5.7-fold higher fluorescence intensity than that in the absence of both inducers. This result confirmed that the assembly of the two LacI operators and the LuxR operator integrated the inputs of IPTG and AHL into the output of GFP transcription. In other words, combination of the two LacI operators and the LuxR operator functioned as a genetic AND gate.

## Discussion

In order to modify the regulatory region of a gene, we here developed a new standard by modifying the BioBricks standard and constructed a new genetic AND gate from standardized biological parts. The Lux-Lac AND gate we constructed is composed of LuxR and LacI operators, whose bindings to the regulatory proteins are controlled by AHL and IPTG, respectively. In order to realize the desired function, our AND gate has two important properties. One is that we prepared standardized parts, each of which has one operator site, and combined them by modular assembly. In contrast, another method of assembly in the synthetic Ara-lac AND gate [10] requires a unique DNA with multiple operator sites for each designed combination of parts. Another important property is that our method is based on the simple mechanism of gene regulation. It would be difficult to simultaneously use several complicated regulation assemblies such as a nonsense-suppression dependent system [11].

Our modification of BioBricks standard allows insertion of parts into an established assembly of other parts. Although In-Fusion BioBrick assembly reported very recently [18] may allow similar insertion of parts with further modifications of their method, it requires primers, each of which covers two parts next to each other in the design of an assembly. This combination-dependent synthesis reduces the utility of the established compatibility of each BioBricks part.

In contrast to design-based strategy we examined in this study, other researchers have used a combinatorial strategy to construct artificial biological devices. In order to establish the fine regulation of a genetic circuit, Guet *et al.* constructed a combinatorial library in which repressor coding sequence and promoters were connected in various orders on a plasmid [19]. By using another combinatorial strategy, Cox III *et al.* constructed several AND gates and OR gates [7]. In their study, operators for LacI, TetR, AraC, or LuxR were randomly inserted to the regulatory region. Though several gates were isolated, several nonfunctional gates were constructed at the same time. The design-based strategy is more effective for creating assemblies since it does not require huge resources. A hybrid strategy in which a combinatorial strategy and our strategy are combined would be effective to implement an artificial biological device with specific quantitative parameter dependence on inputs. In the combinatorial library, a regulatory protein should correspond to multiple operator parts, each of which has a different binding property for the protein.

The number of transcriptional factors and operators used in synthetic biology is increasing, and a combinatorial explosion in the number of possible assemblies must occur in the future. As a method to combat this combinatorial explosion, the design strategy assisted by computer simulations and mathematical models will be effective. Even though the wet-based method has middle-sized parallelism due to the number of cells screened, as indicated by the studies of DNA computer, it would consume a lot of molecules to solve a problem stemming from a combinatorial explosion. The parallelism in computer-based evaluation of assemblies is indeed much lower than that in wet-based evaluation, but the computer-based evaluation time for each assembly will be much shorter in the future. In addition, the activity predicted from computer design often could not correctly reflect the activity of a wet molecule in the present situation. In the future, however, technological advances will reduce the difference between the predicted value and the real value. Thus, computer-assisted rational design will become more effective than the combinatorial strategy.

The genetic logic gates established with our method or its modified version would make the cells recognize the pattern of multiple input-molecules. These engineered cells can be applied in drug-delivery systems, bioremediations, and material-synthesizing systems. A cell that recognizes a lot of inputs replies well to each situation. For example, engineered cells can be designed to release appropriate medicine inside patients upon recognizing tumour markers. Also, the cells that recognize the concentration of pollutants in the environment can be designed to absorb and degrade them. Cells that recognize the coexistence of key intermediates in a metabolic pathway can be designed to optimize the material-synthesizing system. To realize of such a technology, we must prepare a database of regulatory proteins each of which binds both of an input-molecule and an operator. Perhaps the Registry of Standard Biological Parts, which becomes larger and larger every year, is a good candidate database. The Registry of Standard Biological Parts would be more effective than other databases, such as Regulon DB, since the inventory of physical BioBricks parts and information in the database are linked together.

## Conclusions

We implemented a new standard to assemble multiple operator parts in the regulatory region by expanding the BioBricks standard. By assembling LacI and LuxR operator parts with this standardization, we constructed a new genetic AND gate. The easy assembly of multiple operator parts promotes modification of the regulation of gene expression, which is important for reprogramming cellular behavior.

## Methods

### Construction of plasmids

Plasmid pSB-GFP was constructed by introducing *Sal* I, *BamH* I, *Bgl* II, and *Mlu* I restriction sites to BBa_I7100, which was obtained from the Registry of Standard Biological Parts [20]. In order to introduce these sites, PCR mutagenesis was used with the following primers: BglMlu_RBS_plus (5'-GGAGATCTGTCGGATAACGCGTGAGATTAAAGAGGAGAAATACTAGATG-3') and BamSal_pSB3_minus (5 '-GAC AGGATCC AGTCGTC AGTCGACCTCT AGAAGCGGCCGCG-3').

The LacI operator part and Placq were produced by ligation of phosphorylated and annealed oligonucleotides with T4 DNA ligase. The following oligonucleotides were used:

LacI operator part: (5'-GATCGGAATTGTGAGCGGATAACAATTCCAGATCTA TCGTAA-3') and (5'-CGCGTTACGATAGATCTGGAATTGTTATCCGCTCACAATTCC-3');

Placq:(5'-TCGACCGTGACGGATCCTGGTGCAAAACCTTTCGCGGTATGGCATGATAGCGCC-3') and (5'-GATCGGCGCTATCATGCCATACCGCGAAAGGTTTTGCACC AGGATCCGTC ACGG-3').

In order to construct Placq-GFP, a pair of phosphorylated and annealed Placq oligonucleotides was ligated to the pSB-GFP digested with *Sal* I and *Bgl* II. A LuxR operator part was produced by digesting BBF2621, which was also obtained from the Registry of Standard Biological Parts [20]. In order to construct PLuxR-GFP, we prepared a PLuxR fragment containing LuxR expression cassette, a LuxR operator sequence, and luxpR by digesting BBa_F2621 with *Eco*R I and *Spe* I. The fragment was ligated to the pSB-GFP, which was digested with *Eco*R I and *Xba* I. In order to construct Placq-LacI-GFP, Placq-Lacltandem-GFP, and PLuxR-LacItandem-GFP, each pair of the phosphorylated and annealed oligonucleotides was ligated to its parent plasmid digested with *Bgl* II and *Mlu* I.

### Construction of E.coli strain DH5αLacI

The *E. coli* strain DH5αLacI was obtained by transforming DH5α with plasmid pTrc99A [GenBank: U13872], which expresses the LacI repressor.

### Reporter assay

In order to quantitatively determine the performance of the operator parts in the reporter plasmid, the change of fluorescence intensities of the reporter strains in the presence and the absence of the inducer were measured. Overnight cultures of reporter strains grown at 37 °C in LB medium containing appropriate antibiotics were diluted 1:100 in the medium and were incubated at 37 °C as fresh cultures. After their OD_600_ reached 0.6, the fresh cultures were diluted 1:1 in the medium in the presence or absence of various inducers: 1 mM IPTG and/or 4.5 nM N(β-ketocaproyl)-DL-homoserine lactone. After 8-hour incubation at 37 °C, 0.15 ml of each culture was moved to a 96-well plate and its raw fluorescence intensity was measured with a fluorometer (FLA5000, FUJI). The fluorescence intensity was calculated by dividing the measured raw fluorescence intensity by the culture volume and the OD_600_ value. A strain containing the Placq-GFP plasmid, which constitutively expresses GFP, was used as a positive control. A strain containing the pSB-GFP plasmid, which does not express GFP, was used as a negative control.

### Enzymes, media, and chemicals

Restriction enzymes and polynucleotide kinase were purchased from Takara (Shiga, Japan). Pyrobest DNA polymerase for PCR mutagenesis was purchased from Takara (Shiga, Japan). T4 DNA ligase was purchased from Nippon Gene (Tokyo, Japan). DNA sequencing reactions were performed using the BD sequencing kit purchased from Applied Biosystems (CA, USA). All reactions were performed according to the manufacturer's instructions. Synthetic oligonucleotides and sequencing primers were purchased either from Operon (Tokyo, Japan) or Sigma-Aldrich-Japan (Tokyo, Japan). Antibiotics were added to the growth media at the following concentrations: 50 μg/ml ampicillin and 30 μg/ml kanamycin. IPTG and standard chemicals, all of PA grade, were purchased from Nacalai (Kyoto, Japan), and N(β-ketocaproyl)-DL-homoserine lactone was obtained from Sigma-Aldrich Japan.

## List of abbreviations

*E. coli: Escherichia coli*; IPTG: Isopropyl β-D-1-thiogalactopyranoside; AHL: N(β-ketocaproyl)-DL-homoserine lactone; RBS: ribosome-binding site

## Competing interests

The authors declare that they have no competing interests.

## Authors' contributions

SA designed the new standard for assembly of genetic parts. SA, AK, YN, HT, and SH performed the experiments. MU, KY, SM, YS, MH, MY, and DK provided valuable suggestions. SA wrote the manuscript. DK supervised the study and assisted in writing the manuscript.
